# Supplementary material for: Diversity, multifaceted evolution, and facultative saprotrophism in the European Batrachochytrium salamandrivorans epidemic
Source: Nat Commun. 2021 Nov 18;12:6688. doi: 10.1038/s41467-021-27005-0 (PMC8602665; doi:10.1038/s41467-021-27005-0)

Supplementary Information:

Diversity, multifaceted evolution, and facultative saprotrophism in the European *Batrachochytrium salamandrivorans* epidemic

M. Kelly[1*], F. Pasmans[1], J.F. Muñoz[2], T.P. Shea[2], S. Carranza[3], C.A. Cuomo[2+], A. Martel[1+*]

Supplementary Discussions

Assessing phasing: comparing primary and alternative haplotypes

We assessed how effectively Falcon Unzip had phased our *Bsal* assemblies using similar methods to Miller et al. (2018) . In the analysis of phased vs collapsed regions, structural variants between primary and alternative contigs were aligned with MUMmer (NUCmer) and the alignments assessed using Assemblytics[2]. Coverage was assessed using BWA bwasw[3] version 0.7.12 to align PacBio reads and analysed using the Samtools[4] suite version 1.3 and BEDTools[5] version 2.26.0. Alternative and primary contigs were aligned using nucmer from MUMmer[6] v3.22. We found that of the two Falcon Unzip assemblies included in further analysis, the BundBos2013 isolate had a greater proportion of the alternative haplotype assembled (78%) vs the Luik2014 isolate (51%)., this may have been facilitated by the higher variant rate of the BundBos2013 isolate (297,656 SNPs vs 211,536 in the Luik2014 isolate). As predicted, we found the read coverage to be roughly twice as large in collapsed areas as phased regions but found much higher standard variation in coverage, representing the low coverage of genomic regions that were hard to sequence and very high coverage of repetitive regions. The highly repetitive nature of the *Bsal* genome is likely to have contributed to challenges in phasing, with collapsed regions containing roughly twice as many repeats as the phased regions, and on average the GC of the collapsed regions was 4% lower (see SI Table 11). Falcon Unzip has been shown to sometimes falsely recognise highly divergent haplotypes as separate primary contigs, so we aligned those primary contigs with no secondary haplotypes back to all primary contigs using Blastn from BLAST10 v2.2.30 with an e-value of 1e-5 and looked for candidates that aligned to another primary contig for more than 60% of its length but did not find any such candidates. We also looked for structural variants between primary and alternative haplotypes using Assemblytics[2], the results of which are shown in Table 12.

Variant analysis shows two-fold variation in SNP frequency

There is a two-fold variation in the frequency of single nucleotide polymorphisms (SNPs) within the *Bsal* isolates (see Table 13). The BundBos2013 isolate has the highest SNP count and frequency (297,656 SNPs reflecting 7.2 SNPs per kbp) while the Rob2015 isolate has the lowest (136,828 SNPs reflecting 3.6 per kbp). Overall, we found a far lower rate of variance in protein coding regions, with the frequency of SNPs dropping to half that of non-coding regions. Annotation of SNPs with SNPeff indicated that BundBos2013 displayed the largest number of every category of variant, with notably more SNPs predicted to have effects upstream of genes (3.8 times more such annotations as the Rob2015 isolate) but all other isolates contain similar proportions of each variant type (see Table 14).

Bsal genomes are highly repetitive

The first *Bsal* genome[7] found expanded repeat-rich regions compared to non-pathogenic chytrid species. Our long read PacBio data facilitated better resolution of repetitive regions, showing the regions to be even larger than previously thought- making up 38% of the BundBos2013 assembly versus 16% found in Farrer et al. (2017). We found LTR repeats, the most common repetitive element in *Bd*, present in all isolates, making up 1.8-6.2% of the genome. Together with LINEs, representing 3.5-8.1% of the isolate genomes, these made up the most common groups of annotated repeats (see Table 7).

Long read sequencing can also facilitate identification and analysis of larger structural variants (SVs). We used Assemblytics[2] to identify structural variants of size 50-20,000 bp in isolates Luik2014, Captive2015-1, Captive2015-2, Rob2015 and Catalan2018 when aligned to the BundBos2013 assembly. This identified few such variants, with a maximum of 0.3% of an assembly identified as structural variants (in the Catalan2018) when compared to the BundBos2013 assembly. The fragmented nature of our assemblies already led us to assume that such techniques would not identify all structural variants; the conflicts experienced during phylogenetic analysis (see Main text and methods for discussion) indicate possible cryptic recombination, but these are hard to identify from significantly fragmented assemblies. Furthermore, we found the degree of transposable element (TE) expansion in the BundBos2013 and Luik2014 isolates precluded genome alignment in TE heavy regions thus inhibiting the use of such alignment-based methods for SV identification. For example, the nucmer alignment used for Assemblytics analysis of the Captive2015-2 assembly compared to the BundBos2013 assembly (see Methods for details) indicated 32.9Mbp of alignments to 227 of the 233 BundBos2013 contigs, within which Assemblytics identified no minimal tandem (0bp) or repeat (6537bp) contractions. However, 2.4Mbp of the 8.8Mbp (27%) of the BundBos2013 assembly with no alignment to the Captive2015-2 isolate is annotated as either LTR or LINE TEs, compared to 1.5Mbp (4%) of the aligned regions. The Luik2014 assembly also seems to contain these TE expansions, as we see the inverse pattern of TE content in aligned vs unaligned regions (6% of unaligned regions as LINE/LTRs vs 12% of aligned regions), here unaligned regions do not seem repeat-rich in general, with 12.4Mbp of unaligned regions containing 3.8Mbp of repeats - below the overall proportion of repetitive elements in the BundBos2013 assembly (see Table7).

It is well established that many fungal pathogens display “two speed” evolution with areas of low gene density, high repetitive content and low GC content evolving faster than more conserved genome regions[8]. However, when we performed a sliding window analysis of varying window size (5-50kbp) of these three measurements we found no evidence of such genomic islands.

The impact of *Bsal* saprotrophism on mitigation and future considerations

In addition to studies of optimal protocols for eliminating the environmental reservoir of *Bsal*, while minimising environmental damage (for example studying antifungal and disinfection treatments to which *Batrachochytrium* are disproportionately sensitive), the discovery that *Bsal* grows on dead plant material exposes the potential to vector the pathogen into novel environments on plant material.

Trade restrictions on the exportation and importation of salamander species have already been imposed by e.g. the USA[9] and the EU[10] in an effort to reduce the spread and introduction of *Batrachochytrium* species. However, there is a large global trade in aquatic plants, for

example an European and Mediterranean Plant Protection Organization (EPPO) pathway analysis[11] indicated that nearly 5 million aquatic plants were imported into the Netherlands in 2006 alone. While little data was known for where such imports come from, some of the biggest exporters for which data was available included Southeast Asian nations such as Thailand, Singapore and Indonesia, for which *Bsal* prevalence is currently unknown. While the ability of *Bsal* to grow on live aquatic plants is still undetermined, aquatic plant shipments will likely also contain some degree of dead plant material. Furthermore, in contrast to the majority of imported amphibians which are placed in enclosures designed to prevent the escape (and therefore entrance) of amphibian species; a large proportion of aquatic plants are placed into outdoor waterbodies with unrestricted exposure to wild amphibian populations. The cited EPPO analysis determined 90% of imported aquatic plant species represent an invasive species risk due to their exposure to natural waterbodies.

Future mitigation studies should assess the risk such pathways represent- for example by surveying the prevalence of *Bsal* in nations exporting aquatic plants, trying to improve the transparency within the aquatic plant trade and performing e.g. eDNA testing for *Bsal* prevalence in plant shipments. The latter could also be implemented as a preliminary mitigation measure. Furthermore, EPPO already detail hygiene measures and restrictions imposed upon imports of certain plant materials- many of which involve temperature treatments. Given the sensitivity of *Bsal* to temperature[12], further studies could aim to develop similar controls that are endurable for the plant produce but not for *Batrachochytrium* as potential preventative measures.

References

1. Miller, M. E. *et al.* De Novo Assembly and Phasing of Dikaryotic Genomes from Two Isolates of Puccinia coronata f. sp. avenae, the Causal Agent of Oat Crown Rust. *mBio* **9**, e01650-17 (2018).
2. Nattestad, M. & Schatz, M. C. Assemblytics: a web analytics tool for the detection of variants from an assembly. *Bioinformatics* **32**, 3021–3023 (2016).
3. Li, H. & Durbin, R. Fast and accurate long-read alignment with Burrows-Wheeler transform. *Bioinforma. Oxf. Engl.* **26**, 589–595 (2010).
4. Li, H. *et al.* The Sequence Alignment/Map format and SAMtools. *Bioinforma. Oxf. Engl.* **25**, 2078–2079 (2009).
5. Quinlan, A. R. & Hall, I. M. BEDTools: a flexible suite of utilities for comparing genomic features. *Bioinformatics* **26**, 841–842 (2010).
6. Kurtz, S. *et al.* Versatile and open software for comparing large genomes. *Genome Biol.* 9 (2004).
7. Farrer, R. A. *et al.* Genomic innovations linked to infection strategies across emerging pathogenic chytrid fungi. *Nat. Commun.* **8**, 14742 (2017).

8. Möller, M. & Stukenbrock, E. H. Evolution and genome architecture in fungal plant pathogens. *Nat. Rev. Microbiol.* **15**, 756–771 (2017).

9. Listing Salamanders as Injurious Due to Risk of Salamander Chytrid Fungus. https://www.fws.gov/injuriouswildlife/salamanders.html.

10. COMMISSION IMPLEMENTING DECISION (EU) 2018/ 320 - of 28 February 2018 - on certain animal health protection measures for intra-Union trade in salamanders and the introduction into the Union of such animals in relation to the fungus Batrachochytrium salamandrivorans - (notified under document C(2018) 1208). 16.

11. Brunel, S. Pathway analysis: aquatic plants imported in 10 EPPO Countries. *EPPO Bull.* **39**, 201–213 (2009).

12. Beukema, W. *et al.* Microclimate limits thermal behaviour favourable to disease control in a nocturnal amphibian. *Ecol. Lett.* (2020).

13. Fisher, M. C. *et al.* Emerging fungal threats to animal, plant and ecosystem health. *Nature* **484**, 186–194 (2012).

14. Lockhart, S. R. & Guarner, J. Emerging and reemerging fungal infections. *Semin. Diagn. Pathol.* **36**, 177–181 (2019).

15. Fatmi, M. *et al. Pseudomonas syringae Pathovars and Related Pathogens - Identification, Epidemiology and Genomics*. (Springer Science + Business Media, B.V, 2008).

16. World Health Organization, Regional Office for South-East Asia. A brief guide to emerging infectious diseases and zoonoses. WHO Regional Office for South-East Asia. (2014).

17. Bernt, M. *et al.* MITOS: Improved de novo metazoan mitochondrial genome annotation. *Mol. Phylogenet. Evol.* **69**, 313–319 (2013).

Supplementary Information Tables and Figures

		Primary contig length	Number Primary contigs	Primary contig N50	No. Gaps	GC%	Alternative contig length	Number alternative contigs	Alternative contig N50
**BundBos2013**	**PRJNA311566**	32,636,440	5,358	10,506		41			
**BundBos2013**	**Falcon**	**41,552,838**	**227**	**346,311**	**0**	**43**	**36,867,569**	**740**	**100,537**
	HGAP	48,191,222	741	182,497		42.6			
**Luik2014**	**Falcon**	**41,389,572**	**507**	**123,628**	**0**	**44**	**27,675,237**	**1382**	**27,733**
	HGAP	55,297,009	891	132,897		45			
**Catalan2018**	**Flye**	**40,041,066**	**580**	**203,216**	**17**	**42.4**			
	Falcon	27,105,589	375	97,050		42	11,389,915	518	24,989
**Captive2015-1**	HGAP	37,147,929	1,623	59,702					
	**Flye**	**35,589,685**	**1,220**	**79,904**	**6**	**42.3**			
	Falcon	27,465,935	397	97,808		43	16,195,207	741	28,857
**Rob2015**	HGAP	37,038,296	1,189						
	**Flye**	**38,362,267**	**1,253**	**74,857**	**16**	**42.3**			
	Falcon	19,989,825	356	70,519		43	12,851,991	753	21,771
**Captive2015-2**	HGAP	31,156,945	1,299	53,475					
	**Flye**	**34,923,282**	**1,173**	**80,258**	**9**	**42.3**			

Table 1. Genome assembly statistics. Genome assembly summary statistics with published BundBos2013 isolate genome PRJNA311566 (Farrer et al. 2017) for comparison, assemblies highlighted in bold are those included for further analysis.

	BundBos2013	Luik2014	Captive2015-1	Rob2015	Captive2015-2	Catalan2018
**Total proteases count**	1,344	1,475	660	772	590	1060
**M36 metalloprotease Pfam**	202	108	118	148	89	200
**M24 metalloprotease Pfam**	8	15	8	4	5	10
**Metallopeptidase Pfam**	229	135	126	157	97	253
**Total CAZyme count**	399	353	232	248	203	376
**Unique CAZyme hits**	189	207	123	124	128	130
**SigP4 secretory signal**	2,223	2,232	1,518	1,813	1,506	2,331
**SigP4 with Transmembrane signal**	57	87	49	47	38	69
**TMHMM transmembrane domain**	1,598	2,862	1,542	1,643	1,529	1,613
**Crinkler genes**	115	117	111	109	108	115
**Crinklers containing Transmembrane signal**	3	11	2	3	4	3
**Crinklers containing both SigP4 and Transmembrane**	1	1	1	1	1	2
**Crinklers SigP4**	8	8	10	3	6	12

Table 2. Candidate effector proteins Counts of genes on primary contigs predicted to be select effector proteins.

	BundBos 2013	Luik 2014	Captive 2015-1	Rob 2015	Captive 2015-2	Catalan 2018	*Bd* Jel423
**Number of orthogroups containing isolate**	8,318	7,170	8,177	8,293	8,102	8,376	6,115
**Number of orthogroups unique to isolate**	57	909	18	27	22	15	216
**Number proteins in isolate-specific orthogroups**	137	3,398	39	63	49	44	1,782
**Isolate-specific orthogroup genes containing SigP**	13	531	0	0	0	0	406
**Isolate-specific orthogroup genes containing TMHMM**	3	924	0	0	0	0	221
**Isolate-specific orthogroup genes containing Crinklers**	0	75	0	0	0	0	198
**Isolate-specific orthogroup genes containing MEROPs**	11	278	0	0	0	0	141
**Isolate-specific orthogroup genes containing CAZY**	0	46	0	0	0	0	17
**Unassigned:**							
**Number genes unassigned to orthogroups**	575	4,529	291	440	326	151	648
**Unassigned genes containing SigP**	60	319	26	38	30	36	85
**Unassigned genes containing TMHMM**	43	831	30	58	32	12	178
**Unassigned genes containing Crinklers**	7	78	1	2	0	0	6
**Unassigned genes containing MEROPs**	42	214	12	17	6	0	16
**Unassigned genes containing CAZY**	2	32	0	2	2	0	4

Table 3. Summary of OrthoFinder orthogroups or unassigned genes unique to isolates with annotations. Summaries of orthogroups identified by OrthoFinder as specific to one isolate in *Bsal* or *Bd* Jel423.

Species	GenBank Accession Number
Piromyces finnis	GCA_002104945.1
Neocallimastix californiae	GCA_002104975.1
Synchytrium microbalum	GCA_006535985.1
Anaeromyces robustus	GCA_002104895.1
Gonapodya prolifera	GCA_001574975.1
Batrachochytrium dendrobatidis	GCA_000149865.1
Rhizoclosmatium globosum	GCA_002104985.1
Spizellomyces sp. 'palustris'	GCA_006535965.1
Spizellomyces punctatus	GCA_000182565.2
Powellomyces hirtus	GCA_006536005.1
Chytriomyces confervae	GCA_006535975.1
Synchytrium endobioticum	GCA_006535955.1, GCA_006536045.1
Blyttiomyces helices	GCA_003614705.1
Piromyces sp. E2	GCA_002157105.1
Caulochytrium protostelioides	GCA_003615035.1

Table 4. Other sequenced Chytridiomycota species considered in gene family comparisons. Species and GenBank Accession number of Chytridiomycota assemblies checked for the presence of CAZyme family proteins.

CAZyme Family	Other Chytridiomycetes assemblies containing candidates of this family
AA3	*Gonapodya prolifera, Rhizoclosmatium globosum, Spizellomyces palustris, Spizellomyces punctatus, Synchytrium endobioticum, Synchytrium endobioticum (lev), Synchytrium microbalum*
CBM20	*Anaeromyces robustus, Neocallimastix californiae, Piromyces finnis, Piromyces sp E2, Powellomyces hirtus*
CE11	**Batrachochytrium dendrobatidis,** *Anaeromyces robustus, Blyttiomyces helicus, Caulochytrium protosetlioides, Chytriomyces confervae, Gonapodya prolifera, Neocallimastix californiae, Piromyces finnis, Piromyces sp E2, Powellomyces hirtus, Rhizoclosmatium globosum, Spizellomyces palustris, Spizellomyces punctatus, Synchytrium endobioticum, Synchytrium endobioticum (lev), Synchytrium microbalum*
GH3	*Anaeromyces robustus, Chytriomyces confervae, Gonapodya prolifera, Neocallimastix californiae, Piromyces finnis, Piromyces sp E2, Powellomyces hirtus, Rhizoclosmatium globosum, Spizellomyces palustris, Spizellomyces punctatus*
GH89	
GH95	*Anaeromyces robustus, Chytriomyces confervae, Neocallimastix californiae, Piromyces finnis, Piromyces sp E2, Powellomyces hirtus, Rhizoclosmatium globosum, Spizellomyces palustris, Spizellomyces punctatus*
GT13	
GT95	*Spizellomyces palustris, Spizellomyces punctatus*
GT96	*Synchytrium endobioticum, Synchytrium endobioticum (lev), Synchytrium microbalum*

Table 5. Luik2014 "unique" CAZyme families presence in other sequenced Chytridiomycota. Other sequenced Chytridiomycota assemblies (from list in SI Table 4) containing CAZyme families unique to Luik2014 isolate within *Bsal*
165 isolates, closely related *Bd* in bold.

	Number Moving Spores		Number sporangia		Sporangia coverage	
	Incidence Rate Ratios	CI	Incidence Rate Ratios	CI	Estimate	CI
**BundBos2013**	4.17	0.29 – 60.84	51.1	33.89 – 78.63	0.14	0.07 – 0.29
**Luik2014**	3.02	0.16 – 56.17	1.75	1.13 – 2.70	1	0.39 – 2.57
**Captive2015-2**	0.05	0.00 – 3.09	0.23	0.14 – 0.35	0.18	0.04 – 0.77
**Captive2015-1**	0.37	0.02 – 8.40	0.52	0.34 – 0.81	0.11	0.03 – 0.45
**Rob2015**	2.87E+08	0.00 – Inf	0.82	0.53 – 1.28	0.17	0.05 – 0.54
**MediumTGhL-control**	9.93	0.65 – 152.90	1.79	1.16 – 2.76	0.32	0.12 – 0.84
**Day.1**	0.72	0.47 – 1.12	1.28	1.23 – 1.33	0.99	0.90 – 1.09
**Luik2014:MediumTGhL-control**	0.06	0.00 – 1.22	0.26	0.14 – 0.48	0.82	0.20 – 3.32
**Captive2015-2:MediumTGhL-control**	12.99	0.21 – 800.04	4.52	2.44 – 8.38	6.58	1.17 – 36.99
**Captive2015-1:MediumTGhL-control**	0.94	0.04 – 23.58	2	1.08 – 3.70	28.92	5.55 – 150.77
**Rob2015:MediumTGhL-control**	0	0.00 – Inf	1.09	0.59 – 2.02	14.57	3.39 – 62.60
**Luik2014:Day.1**	1.09	0.68 – 1.74			1.12	0.99 – 1.26
**Captive2015-2:Day.1**	1.5	0.85 – 2.64			1.04	0.87 – 1.24
**Captive2015-1:Day.1**	1.34	0.83 – 2.18			1.16	0.98 – 1.37
**Rob2015:Day.1**	0.03	0.00 – Inf			1.24	1.08 – 1.42
**MediumTGhL-control:Day.1**	1.21	0.78 – 1.88			1.31	1.17 – 1.48
**Luik2014:MediumTGhL-control:Day.1**	1.09	0.67 – 1.76			0.82	0.70 – 0.97
**Captive2015-2:MediumTGhL-control:Day.1**	0.76	0.43 – 1.35			0.87	0.70 – 1.08
**Captive2015-1:MediumTGhL-control:Day.1**	0.87	0.53 – 1.42			0.71	0.58 – 0.87
**Rob2015:MediumTGhL-control:Day.1**	28.37	0.00 – Inf			0.69	0.58 – 0.82

Table 6. Estimates and standard errors from lima bean growth experiments. Negative binomial generalised linear models were fit to motile spores and sporangia count data, a beta regression model best fit sporangia coverage That spore count showed no significant interaction with any isolate, medium or time shows that it may not be a suitable measurement of growth- this may be because a high motile spore count can represent both a healthy culture that is growing well with mature sporangia producing a lot of new spores, or it can represent a failure of spores to attach and mature into sporangia- thus representing a poorly growing culture.

	BundBos2013		Luik2014		Captive2015-1		Captive2015-2		Rob2015		Catalan2018	
	bp	%	bp	%	bp	%	bp	%	bp	%	bp	%
**Repeat bases**	16208475	39	11859960	29	8112325	23	7166148	21	10095640	26	12932420	32.3
**SINEs:**	0	0	76229	0.2	0	0	0	0	0	0	0	0
**LINEs:**	1469613	3.5	3360061	8.1	448150	1.3	490030	1.4	1303233	3.4	2663328	6.65
**LINE1**	55667	0.1	56164	0.1	224919	0.6	19774	0.1	0	0	0	0
**LINE2**	71406	0.2	24366	0.1	0	0	0	0	0	0	0	0
**L3/CR1**	134031	0.3	0	0	0	0	0	0	0	0	0	0
**LTR elements:**	3270277	7.8	2554916	6.2	1425631	4	242193	0.7	800677	2.1	1627843	4.07
**ERV_classI**	8765	0	0	0	23291	0.1	0	0	0	0	37866	0.09
**ERV_classII**	20581	0.1	0	0	0	0	0	0	0	0	0	0
**DNA elements:**	1453971	3.5	833628	2	1215579	3.4	551745	1.6	1316813	3.4	2169530	5.42
**Unclassified:**	9150686	22	4565706	11	4504149	13	5056950	15	6006158	16	5842891	14.59
**Total interspersed repeats:**	15344547	37	11390540	27	7593509	21	6340918	18	9426881	25	12303592	30.73
**Small RNA:**	0	0	0	0	0	0	0	0	41449	0.1	93652	0.23
**Satellites:**	42	0	64	0	64	0	279981	0.8	128	0	64	0
**Simple repeats:**	717939	1.7	330998	0.8	337707	1	351108	1	421358	1.1	352321	0.88
**Low complexity:**	146186	0.4	139382	0.3	181045	0.5	194141	0.6	205916	0.5	185145	0.46
**Insertion Total**			321	0.0	208	0.0	212	0.0	3000	0.0	584	0.0
**50-500bp**			321	0.0	208	0.0	212	0.0	57	0.0	584	0.0
**500-10,000bp**			0	0.0	0	0.0	0	0.0	2943	0.0	0	0.0
**10,000-20,000bp**			0	0.0	0	0.0	0	0.0	0	0.0	0	0.0
**Deletion Total**			2531	0.0	4886	0.0	4439	0.0	2587	0.0	3279	0.0
**50-500bp**			117	0.0	178	0.0	167	0.0	327	0.0	1019	0.0
**500-10,000bp**			2414	0.0	4708	0.0	4272	0.0	2260	0.0	2260	0.0
**10,000-20,000bp**			0	0.0	0	0.0	0	0.0	0	0.0	0	0.0
**Tandem expansion Total**			87108	0.2	0	0.0	17335	0.0	0	0.0	36963	0.1
**50-500bp**			93	0.0	0	0.0	0	0.0	0	0.0	0	0.0
**500-10,000bp**			29535	0.1	0	0.0	5462	0.0	0	0.0	26212	0.1
**10,000-20,000bp**			57480	0.1	0	0.0	11873	0.0	0	0.0	10751	0.0
**Tandem contraction Total**			0	0.0	4666	0.0	0	0.0	5991	0.0	0	0.0
**50-500bp**			0	0.0	0	0.0	0	0.0	0	0.0	0	0.0
**500-10,000bp**			0	0.0	4666	0.0	0	0.0	5991	0.0	0	0.0
**10,000-20,000bp**			0	0.0	0	0.0	0	0.0	0	0.0	0	0.0
**Repeat expansion Total**			187	0.0	7145	0.0	2405	0.0	375	0.0	33244	0.1
**50-500bp**			187	0.0	0	0.0	67	0.0	375	0.0	0	0.0
**500-10,000bp**			0	0.0	7145	0.0	2338	0.0	0	0.0	3422	0.0
**10,000-20,000bp**			0	0.0	0	0.0	0	0.0	0	0.0	29822	0.1
**Repeat contraction Total**			9033	0.0	2823	0.0	6537	0.0	11900	0.0	56716	0.1
**50-500bp**			0	0.0	0	0.0	0	0.0	53	0.0	260	0.0
**500-10,000bp**			9033	0.0	2823	0.0	6537	0.0	11847	0.0	13569	0.0
**10,000-20,000bp**			0	0.0	0	0.0	0	0.0	0	0.0	42887	0.1
**Total all SV Types**			99180	0.2	19728	0.1	30928	0.1	23853	0.1	130786	0.3

Assemblytics alignment summaries:

	**Luik2014**	**Captive2015-1**	**Captive2015-2**	**Rob2015**	**Catalan2018**
**Aligned regions (Mbp)**	29.3	34	32.9	35.1	38.1
**Number of contigs with alignment**	232	232	227	229	232
**Unaligned regions (Mbp)**	12.4	7.74	8.84	6.64	3.64
**Aligned region annotated as TE (Mbp)**	3.41	1.91	1.52	2.21	2.94
**Aligned region annotated as TE (%)**	11.6	5.62	4.62	6.30	7.72
**Unaligned region annotated as TE (Mbp)**	0.86	1.95	2.31	1.71	1.03
**Unaligned region annotated as TE (%)**	6.91	25.2	26.1	25.8	28.3

Table 7. Summary of isolate assembly repetitive elements and structural variants. Summary of repetitive elements identified by RepeatMasker following RepeatModeller (categories listed in bold) and summary of structural variants (SVs) identified by Assemblytics from alignment of isolate assembly to BundBos2013 assembly, annotated by size of SV (categories listed in italics). Sequences were aligned as per Assemblytics requirements (see methods), summary of alignments and LTR or LINE Transposable Element (TE) composition in bottom 8 rows (to 3 s.f.).

	Protein Number (primary contigs)	coding sequence length	% coding sequence	BUSCO score %
BundBos2013 PRJNA311566	10,138			71.30
**BundBos2013 Falcon**	**12,269**	**22,553,210**	**54.03**	**96.6**
**Luik2014 Falcon**	**17,091**	**26,630,348**	**64.07**	**93.5**
**Captive2015-1 Flye**	**10,482**	**21,039,186**	**59.1**	**94.1**
**Rob2015 Flye**	**11,444**	**21,938,479**	**57.2**	**93.8**
**Captive2015-2 Flye**	**10,353**	**20,612,907**	**59.0**	**91.0**
**Catalan2018 Flye**	**11,289**	**23,622,120**	**59.0**	**97.6**
Bd Jel423	9,879			
Captive2015-1 Falcon	9,852	15,907,934	58.69	63.4
Rob2015 Falcon	8,671	15,497,041	56.38	71.7
Captive2015-2 Falcon	6,247	11,559,360	57.83	67.2

Table 8. Assembly protein annotation statistics. Assemblies highlighted in bold are those included in this manuscript, with prior published BundBos2013 isolate genome PRJNA311566 (Farrer et al. 2017) and *Bd* Jel423 (PRJNA13653, GCA_000149865.1) included for comparison.

	Number of Reads	Average read length (bp)	Maximum length (bp)	Sample yield (MB)
**BundBos2013**	1,094,387	5,935	60,944	5,881
**Luik2014**	991,001	6,301	71,207	6,896
**Captive2015-1**	885,122	3,901	82,403	3,453
**Rob2015**	522,501	4,754	58,587	2,484
**Captive2015-2**	1,009,137	3,467	57,930	3,467
**Catalan2018**	1,209,057	7,885	92,434	9,534

Table 9. Read Information from PacBio sequencing

First subculture			Second subculture			Third subculture			Subsequent subcultures		
**LB**	**TGhL**	**AD**	**LB**	**TGhL**	**AD**	**LB**	**TGhL**	**AD**	**LB**	**TGhL**	**AD**
4	0	5	4	0	5	4	0	5	4	0	5
3	0	6	3	0	6	3	0	6	3	0	6
2	2	5	2	1	7	2	1	7			
2	1	6	2	1	7	2	1	7			
1	2	6	2	1	7	2	1	7			
0	9	0	0	9	0	0	9	0			

Table 10. **Long term culture in Lima bean medium, medium compositions.** Components of media in millilitres, LB = lima bean media, TGhL = tryptone-gelatin hydrolysate-lactose , AD = sterile distilled water

		BundBos2013	Luik2014
**Length**	Total	41,738,742	41,561,795
	Haplotyped	32,897,204	21,394,160
	Collapsed	8,841,538	20,167,635
	Proportion phased	0.788169514	0.514755438
**Coverage**	Assembly mean	23	58
	Haplotyped- mean	54.91	38.85
	Haplotyped- standard deviation	43.8	34.8
	Collapsed-mean	96.73	59.32
	Collapsed- standard deviation	119.9	71.21
**Repeats**	Average	28,209,931 bp	20,777,498 bp
		(35.89 %)	(30.02 %)
	Haplotyped	0.313214283	0.306480513
	Collapsed	0.682513603	0.248835683
**GC**	Haplotyped	0.380864	0.348224
	Collapsed	0.348109	0.343851
**Variants**	Haplotyped-number	244,975	178,139
	Haplotyped-frequency (per kbp)	7.446681487	8.326524622
	Collapsed-number	82,839	65,818
	Collapsed-frequency (per kbp)	9.369297514	3.263545775

Table 11. Assessing phased annotations. Comparing length, coverage, GC content, repetitive regions and variant content of haplotyped and non-phased (collapsed) regions across the two Falcon Unzip assemblies included in our analyses

		BundBos2013			Luik2014		
		Count	Total bp	% Prim Contigs	Count	Total bp	% Prim Contigs
**Insertion**	50-500 bp:	86	10,256	0.024571895	47	5,141	0.012369533
	500-10,000 bp:	60	172,219	0.412611861	25	64,161	0.154374949
	Total:	146	182,475	0.437183756	72	69,302	0.166744483
**Deletion**	50-500 bp:	95	11,153	0.026720978	38	3,983	0.00958332
	500-10,000 bp:	68	227,971	0.546185604	33	100,504	0.241818237
	Total:	163	239,124	0.572906582	71	104,487	0.251401558
**Tandem expansion**	50-500 bp:	14	2,972	0.007120483	10	1,920	0.004619627
	500-10,000 bp:	59	300,920	0.720960876	32	178,909	0.430465046
	Total:	73	303,892	0.728081359	42	180,829	0.435084673
**Tandem contraction**	50-500 bp:	15	3,271	0.007836844	15	2,488	0.005986267
	500-10,000 bp:	26	120,764	0.289333109	12	3,357	0.008077129
	Total:	41	124,035	0.297169953	27	36,058	0.086757562
**Repeat expansion**	50-500 bp:	27	5,053	0.012106258	18	3,997	0.009617005
	500-10,000 bp:	35	133,435	0.319690996	11	41,268	0.099293113
	Total:	62	138,488	0.331797254	29	45,265	0.108910118
**Repeat contraction**	50-500 bp:	34	6,087	0.014583573	18	2,695	0.006484321
	500-10,000 bp:	48	195,333	0.467989668	21	94,881	0.228288985
	Total:	82	20,142	0.048257324	39	97,576	0.234773306
**Total**	Total all variants	567	1,189,434	2.849712145	280	533,517	1.283671699

Table 12. Assemblytics genome rearrangement annotations of falcon phased assemblies. Comparing alternative and primary contigs for genomic rearrangements. We see higher rates of structural variants between primary and alternative contigs in the Luik2014 isolate than when comparing the Luik2014 isolate aligned to the BundBos2013 isolate (supplementary Table 7)

	PB SNP number	Freq per kbp	Read-based heterozygosity	PB and Samtools high conf. subset	SNP frequency in coding sequence	SNP frequency in non-coding sequence	GATK Illumina SNP number
**BundBos2013**	297,656	7.2	0.7	201,180	1.9	4.28	313,693
**BundBos2018** -time series pair							315,361
**Luik2014**	211,536	5.11	0.685	119,076	1.92	5.39	294,884
**Luik2017** -time series pair							298,951
**Rob2014** -time series pair							323,642
**Rob2015**	136,818	3.6	0.655	75,850	1.34	2.84	330330
**Captive2015-2**	146,913	4.2	0.594	79,339	1.45	3.46	354,975
**Captive2015-1**	165,410	4.6	0.665	89,471	1.54	3.93	357,328
**Catalan2018**	231,428	5.8	0.8	170,087	3.2	5.7	362,627

Table 13. Variant statistics for primary contigs of isolate assemblies; PB = PacBio SMRT Link Variant Caller, Samtools = identified using Samtools mpileup, other than “GATK Illumina SNP number” row, all rows refer to PacBio data aligned back to assembly primary contigs.

SNPeff annotation	BundBos 2013	Luik2014	Captive2015-1	Rob2015	Captive2015-2	Catalan2018
**Downstream**	27,865	20,511	10,983	8,252	8,908	45,120
**Intergenic**	2,608	2,344	1,193	1,032	689	11,934
**Intron**	1,379	543	671	519	386	8,648
**Non-synonymous coding**	24,798	18,645	9,498	8,526	8,658	4,368
**Non-synonymous start**	10	6	6	5	1	
**Splice site acceptor + intron**	213	170	69	48	67	7
**Splice site donor + intron**	154	139	61	45	50	6
**Start lost**	60	34	43	32	28	2
**Stop gained**	417	354	238	226	213	54
**Stop lost**	123	139	57	53	54	8
**Synonymous coding**	55,014	38,907	8,623	8,033	7,994	8,484
**Synonymous stop**	45	39	23	25	25	8
**Upstream**	186,535	129,658	58,006	49,054	52,266	46,040

Table 14. SNPeff annotation. Counts of SNPs categorised by SNPeff predicted function effect

Supplementary Figures

Figure 1. Number of genome assemblies in fungal and bacterial species. Scatterplot of the number of genome assemblies present on NCBI per species (as of 20th January 2020), comparing fungal and bacterial species. Triangle points represent the 50 species with the most assemblies from each Kingdom, circular points represent a select group of high interest and emerging pathogens from the literature[13–16] with the number of assemblies and species name labelled alongside the data point for these high interest species.

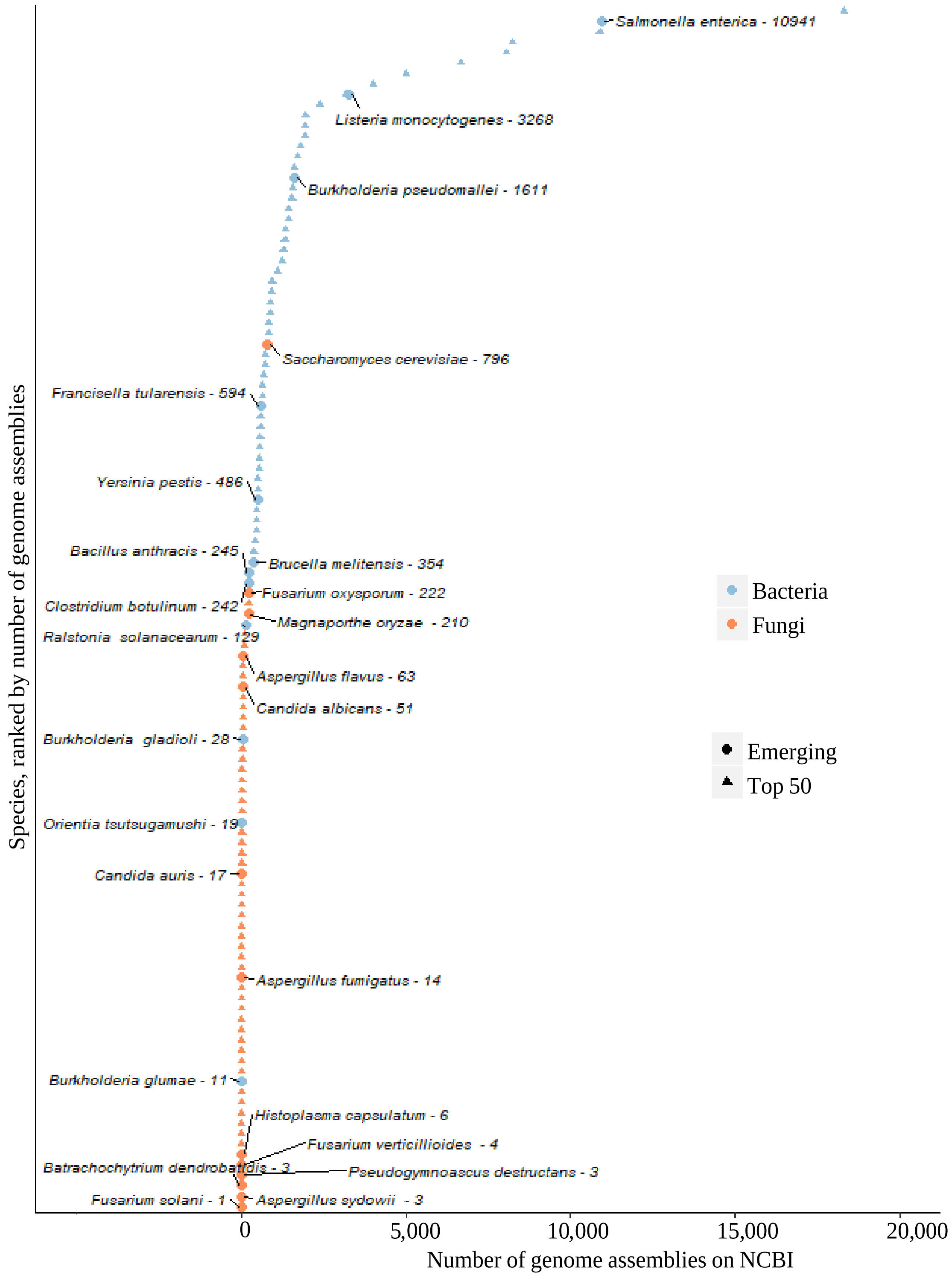

Figure 2. Clustering of isolates from the same outbreak site: isolate outbreak variation and locations. A) Pairwise comparison of the number of "private" variants (indels and SNPs) present in only one of the isolate pairs for all possible pairings- triangles indicate isolate pairs from the same outbreak site, showing lower variant counts, and the distribution of other counts in pairs illustrate the similarity when comparing e.g. both BundBos2013 and BundBos2018 vs other isolates **B)** Principle Component Analysis of SNP variants for all isolates- isolates of the same colour are collected from the same site and seen to cluster together, Captive 2015-1 and Captive2015-2 isolates were collected from outbreaks in two separate captive populations **C)** Map of locations of isolations from wild outbreaks, with year of isolation also noted in the legend. Locations of isolations from captive populations (Captive2015-1 and Captive2015-2) are not provided to maintain anonymity.

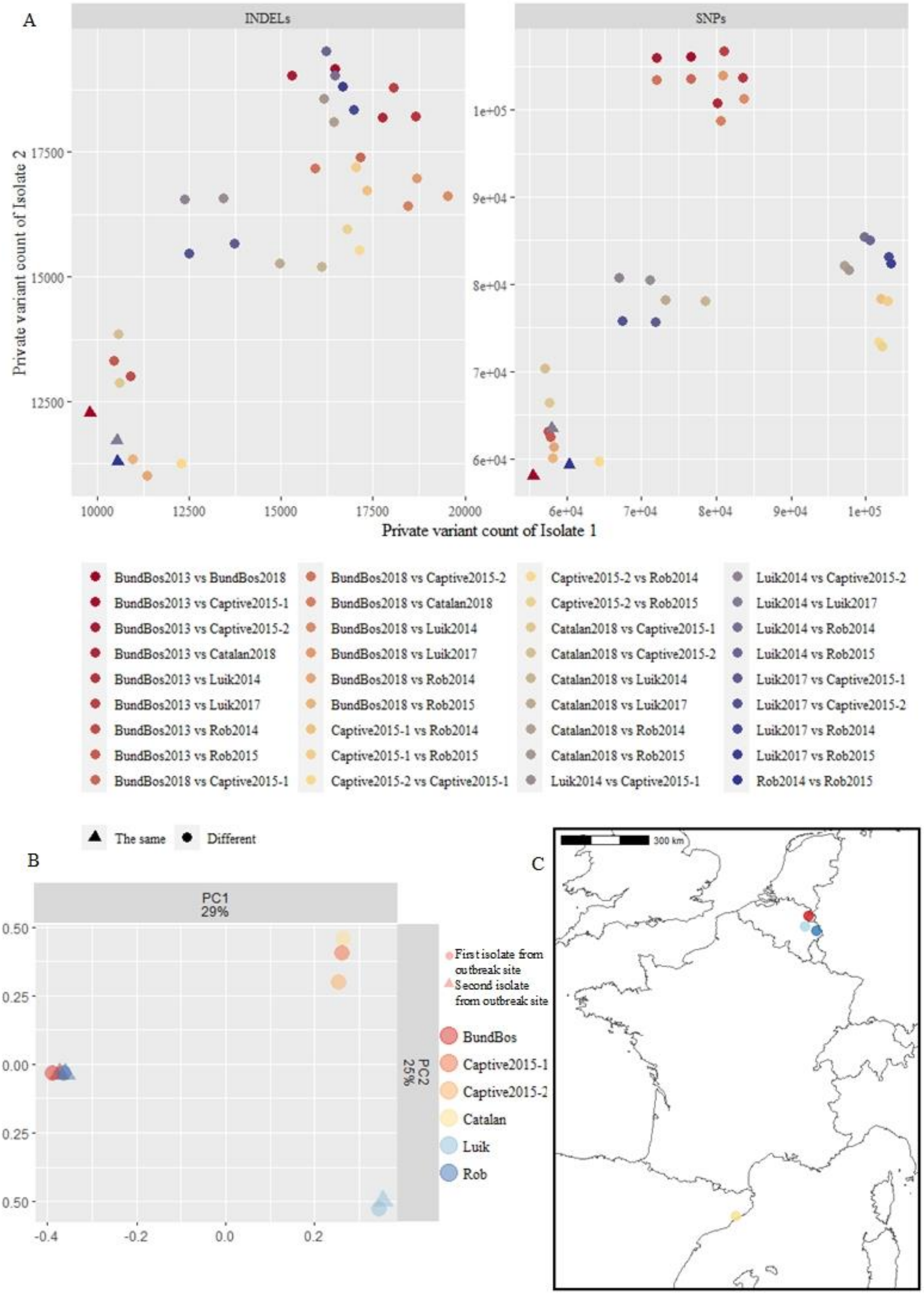

Figure 3. Illumina SNP-based and PacBio OrthoFinder core ortholog phylogeny with *Bd* Jel423 as an outgroup. A) OrthoFinder inferred phylogeny based on the trees of 4,073 orthogroups containing genes from all *Bsal* isolates and *Bd* Jel423 assembly (PRJNA13653, GCA_000149865.1). **B)** Identity by Descent dendrogram inferred using SNPrelate hclust() and ibs() functions on 8,539 SNPs (for more details see Methods) **C)** Beast2 SNAPP dated phylogeny inferred using 8,539 SNPs in linkage equilibrium, node labels represent median height (years), with 95% HPD intervals in brackets, and node posterior support noted in italics

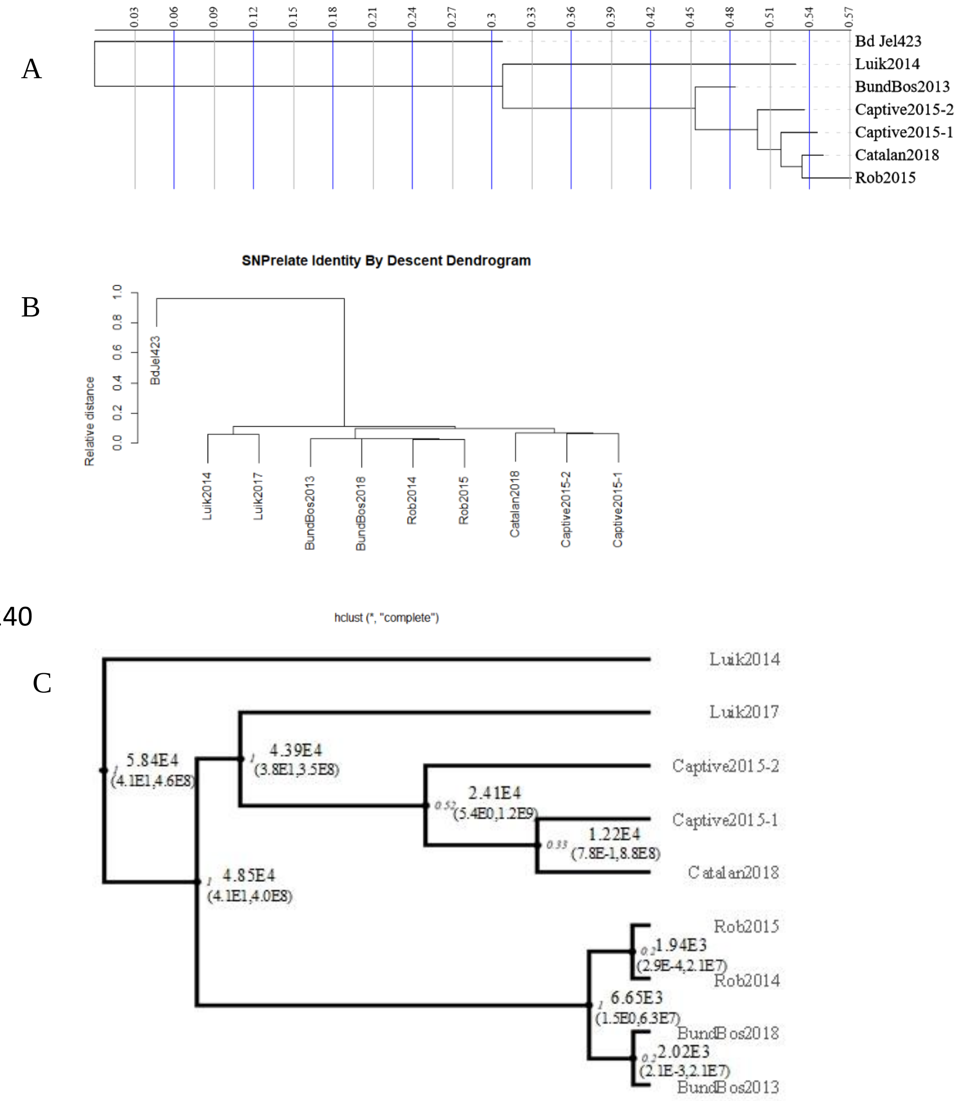

Figure 4. Sequenced Chytridiomycetes assemblies. Chytridiomycetes genome assembly sizes: bar height represents genome assembly size (Mbp), yellow scatterdiagram points represent number of proteins (per 1000), orange bars indicate assemblies from this study, blue bars indicate assemblies from previous studies available on NCBI.

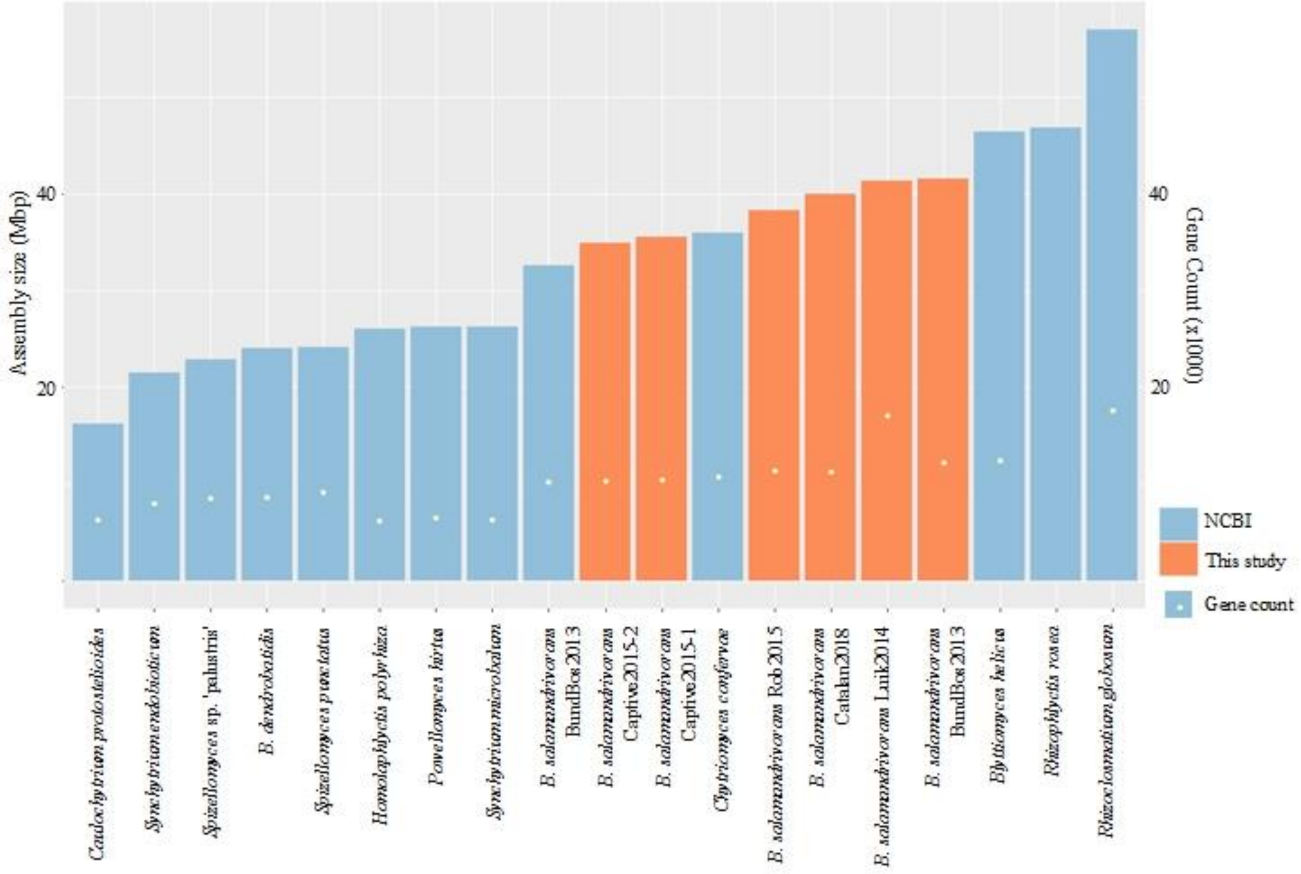

Figure 5. **CAZyme chitin metabolism family comparisons.** Gene copy numbers per isolate of genes predicted to be involved in chitin metabolism

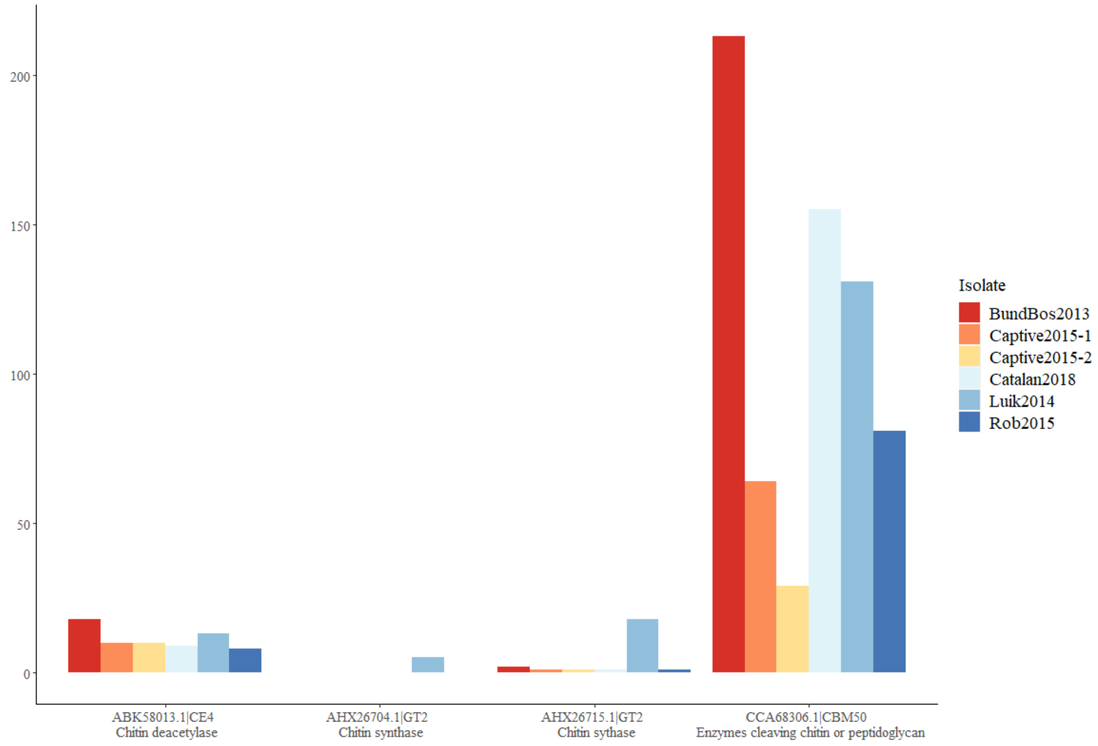

Figure 6. M36 metalloprotease family gene tree. FastTree inferred gene tree of M36 Metalloprotease candidates, a family of proteases similar to Aspergillus fungalysin and associated with host invasion in other host - fungal pathogen systems. These appear to exhibit both pre- and post- divergence expansions with multiple clades containing genes from all isolates, but also expansion in the BundBos2013 isolate which contains 73-132 more M36 metalloprotease genes than the other isolates. However, these expansions are seen as duplications within highly numerous clades, rather than massive expansions within a few clades as seen in the S8A serine peptidases (Fig 2c).

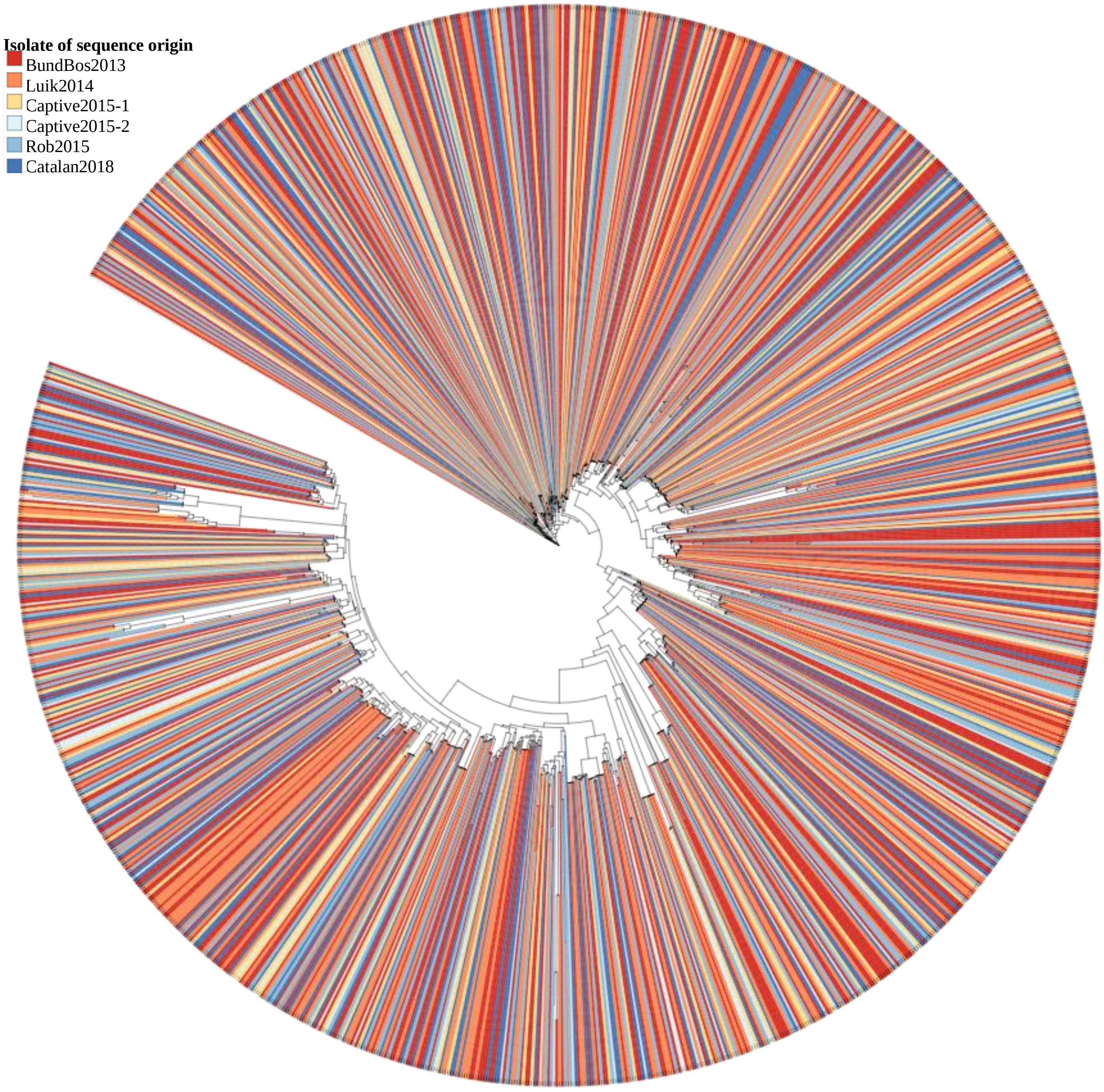

Figure 7. Lima bean vs TGhL growth plots. Boxplots of other growth metrics for each isolate in vegetative Lima Bean (LB) medium (red boxes) and standard TGhL broth (blue boxes) at days 5 and 10 after subculture. *n* = 60 (3 replicates per treatment per isolate per experiment, repeated for 2 experiments). Boxplots constructed with the centre representing the median, the bounds of the box representing 25th and 75th percentiles, whiskers representing the biggest or smallest value within 1.5 * interquartile range of 25th/75th percentiles and data points outside this as outlier points. A) number of motile spores per 20x field of vision. We see higher spore counts with TGhL for all isolates. We see higher spore counts at 5 days than 10 days for many isolates as spore count increases with sporulation, but decreases as spores mature into sporangia. B) proportion of 20x field of vision covered by mature sporangia., for all isolates and conditions this increases over time, with only the Luik2014 isolate showing higher sporangia coverage with the LB medium than standard TGhL medium.

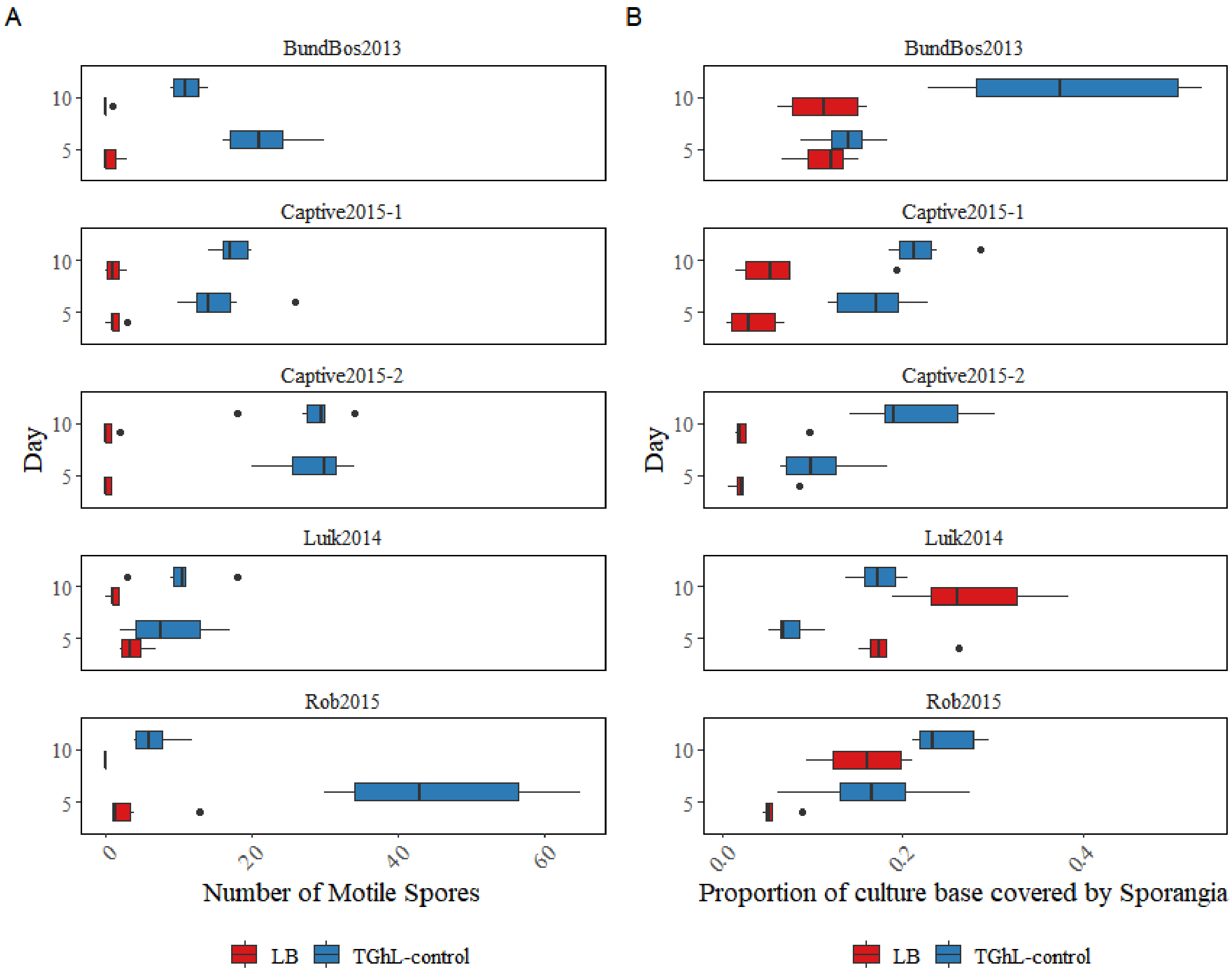

Figure 8. Incidence Rate Ratio estimates and confidence intervals from truncated negative binomial hurdle model fit to sporangia counts of growth of five isolates on autoclaved hay, red line indicates a ratio of 1 i.e. no different from intercept. Blue dots represents IRR estimate with 95% confidence interval lines, calculated as exp(log(IRR estimate) +/- 1.96*standard error (SE) of log(IRR)).

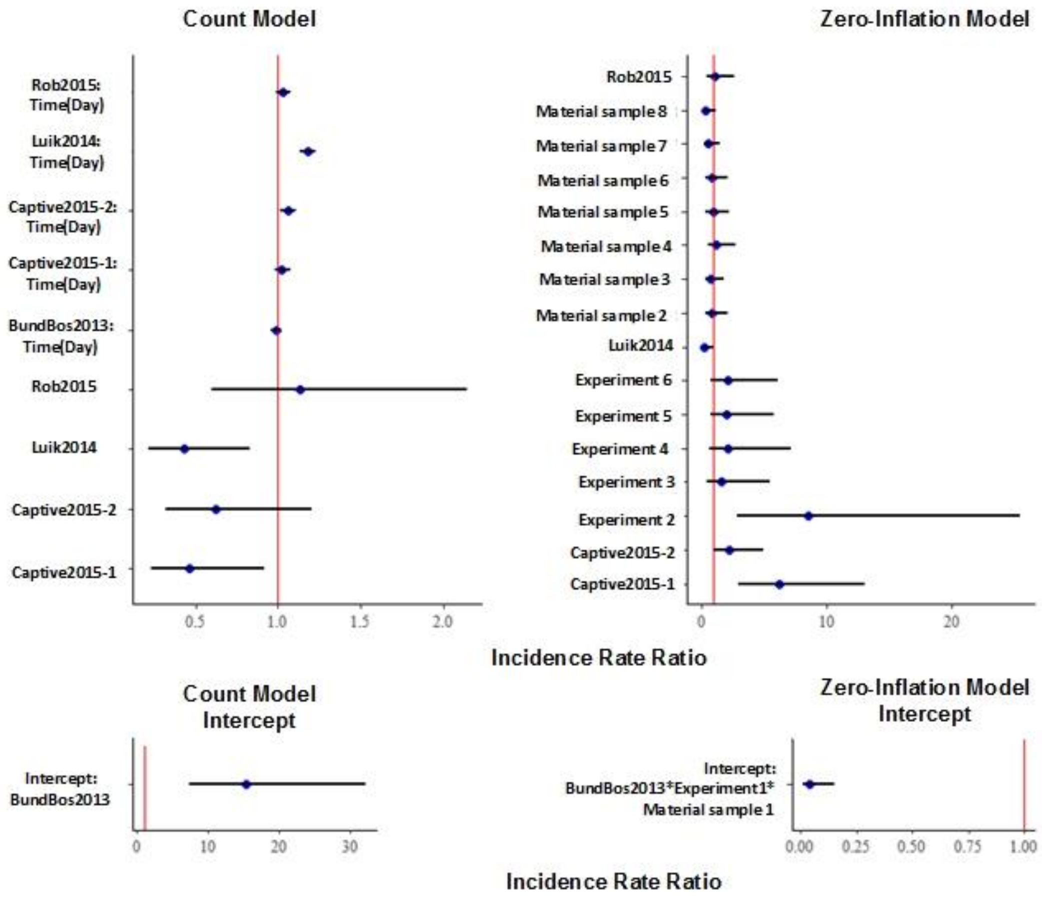

Figure 9. Incidence Rate Ratio estimates and confidence intervals from (A) the zero-inflated negative binomial model fit to sporangia growth counts of the time-series pair of isolates BundBos2013 and BundBos2018 on autoclaved hay, n = 72 (12 independent samples per isolate per experiment, repeated for 3 independent experiments) and (B) the truncated negative binomial hurdle model fit to sporangia counts of growth of two isolates (BundBos2018 and Luik2014) on autoclaved beech leaf litter, n = 20 (5 independent samples per isolate per experiment, repeated for 2 independent experiments), red line indicates a ratio of 1 i.e. no different from the intercept or null hypothesis in the case of the intercept. Blue dote represents IRR estimate with 95% Confidence interval lines, 95% confidence interval lines, calculated as exp(log(IRR estimate) +/- 1.96*standard error (SE) of log(IRR)).)

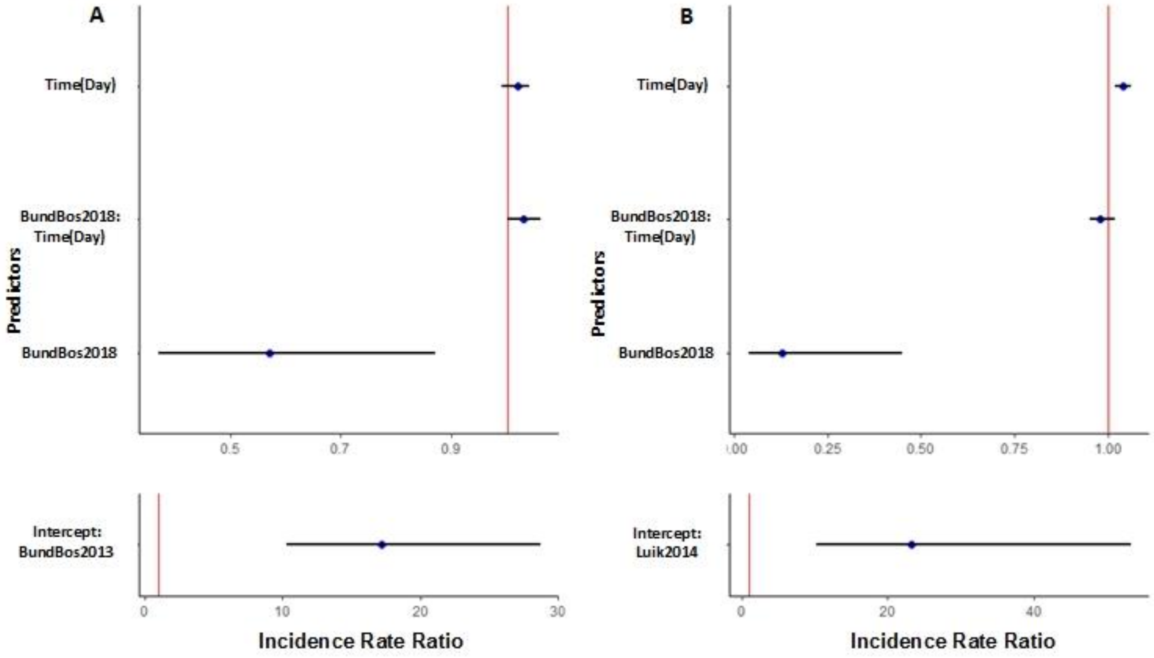

SI Figure 10. Pairwise comparisons of gene presence/absence and sequence deletion/duplication data from CNVnator. Illumina sequence reads from isolate pairs from wild outbreaks BundBos, Rob and Luik were aligned to both the BundBos2013 assembly and the assembly from the outbreak from which they were isolated (Luik2014 or Rob2015 assemblies). Scatter plots show pairwise comparisons of total size and number of genes differentially deleted or duplicated as identified by CNVnator – i.e. the total size of the sequence or number of genes identified as deleted in Isolate 1 but not in the other isolate in the pair comparison. Points are coloured according to the identity of Isolate 1 and circular points represent a comparison with an isolate from a different outbreak site whereas triangular points represent a comparison with the other isolate from the same outbreak site. A) represents deletions identified when all isolate Illumina reads are aligned to the BundBos2013 assembly. B) represents deletions identified when Illumina reads from Luik2014 and Luik2017 are aligned to the Luik2014 assembly, Illumina reads from Rob2014 and Rob2015 are aligned to the Rob2015 assembly and Illumina reads from BundBos2013 and BundBos2018 are aligned to the BundBos2013 assembly. C) represents duplications identified when all isolate Illumina reads are aligned to the BundBos2013 assembly. D) represents duplications identified when Illumina reads from Luik2014 and Luik2017 are aligned to the Luik2014 assembly, Illumina reads from Rob2014 and Rob2015 are aligned to the Rob2015 assembly and Illumina reads from BundBos2013 and BundBos2018 are aligned to the BundBos2013 assembly.

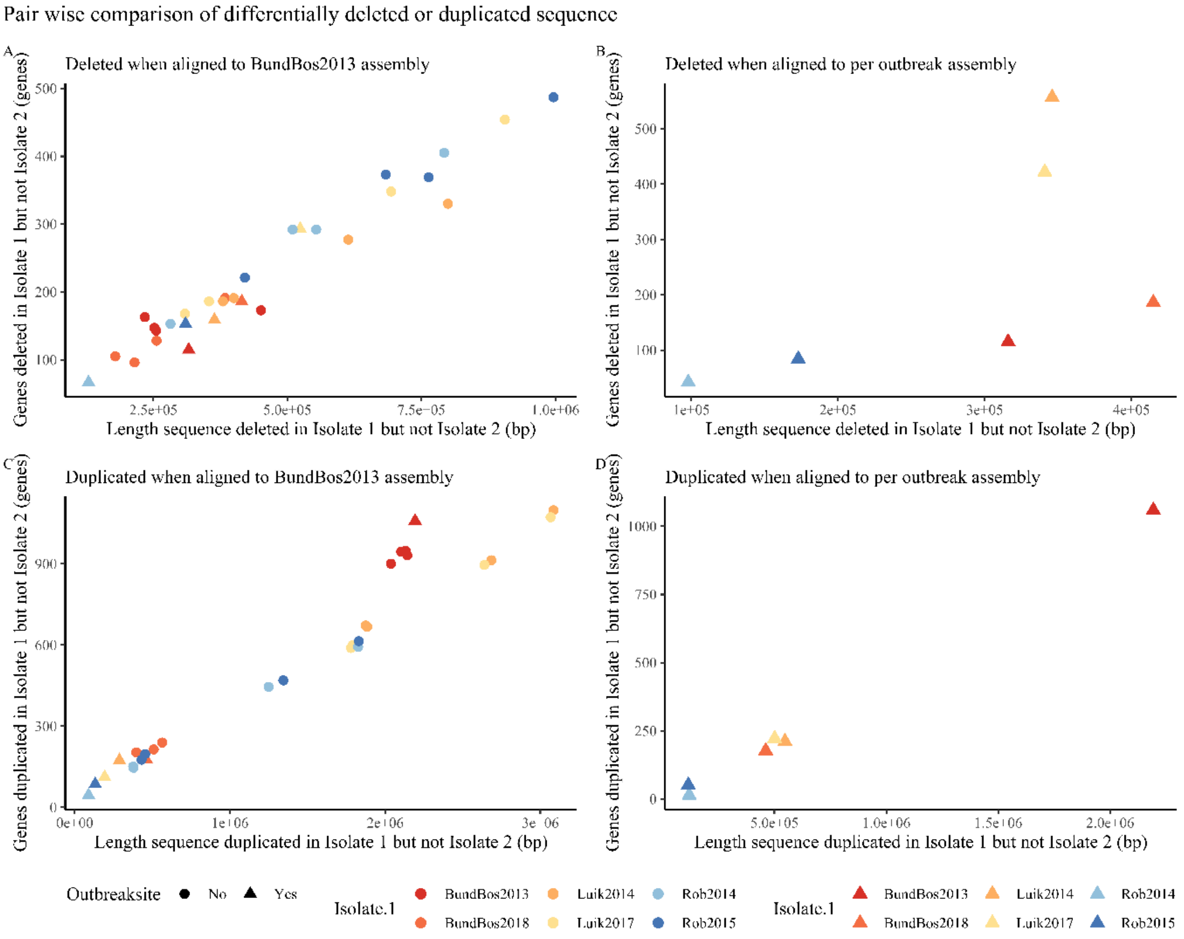

Figure 11. Acetyl xylan esterases (AXE1) Fasttree gene tree. Sequence from Luik2014 isolate indicated with black name label and blue triangle in gene tree generated from 5619 sequences identified as AXE1 on uniprot (downloaded 29th January 2020) and two Luik2014 isolate candidate HGT sequences.

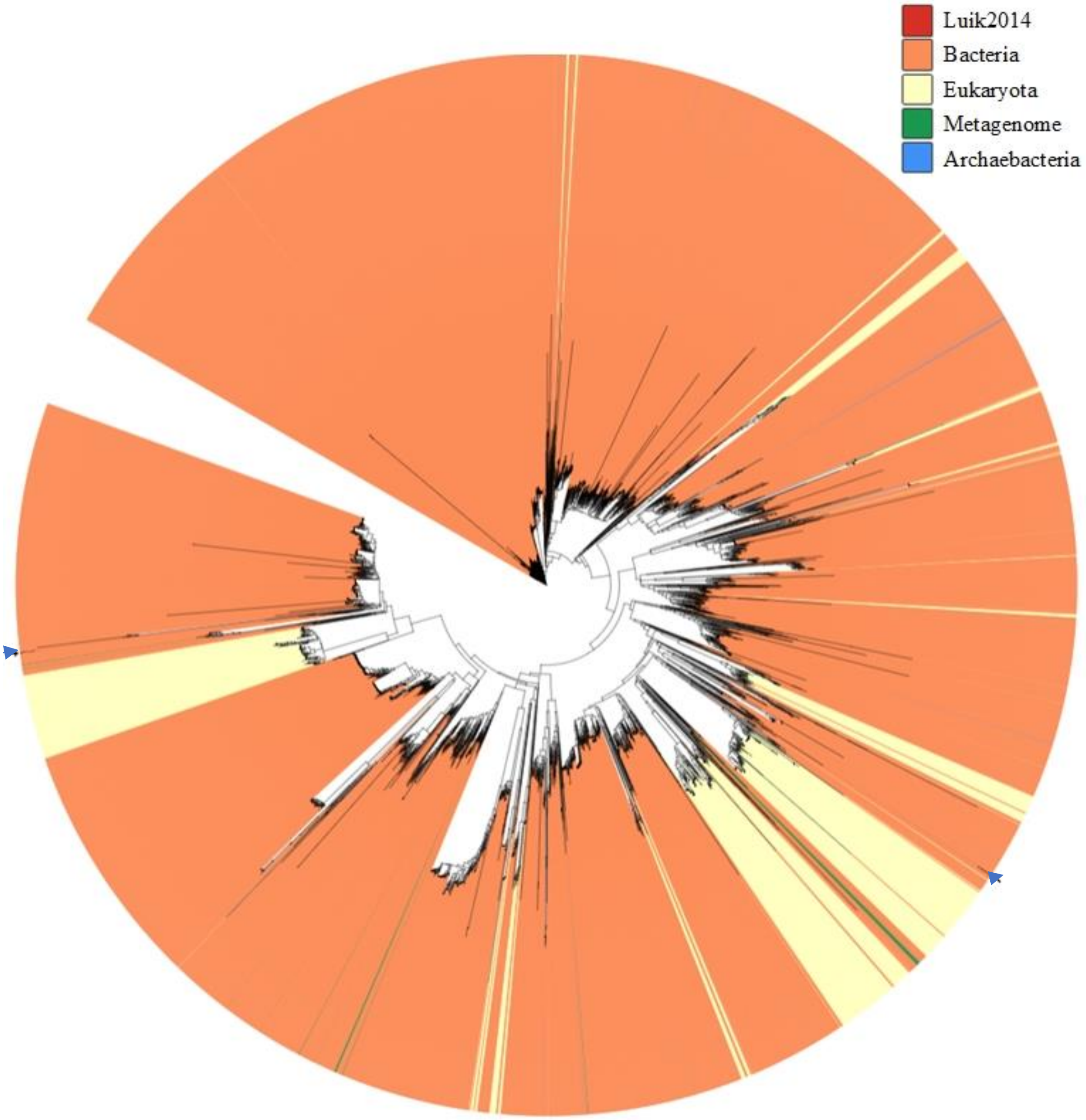

Figure 12. Genome assembly content from different assemblers. Scatterplot of repetitive content, protein-coding content length and protein count from assemblies constructed using HGAP, Flye and Falcon-Unzip. Although our genome assemblies are fragmented, the same patterns of genome content (e.g. comparatively higher repetitive content in BundBos2013, comparatively higher protein-coding content in Luik2014) hold across assemblies.

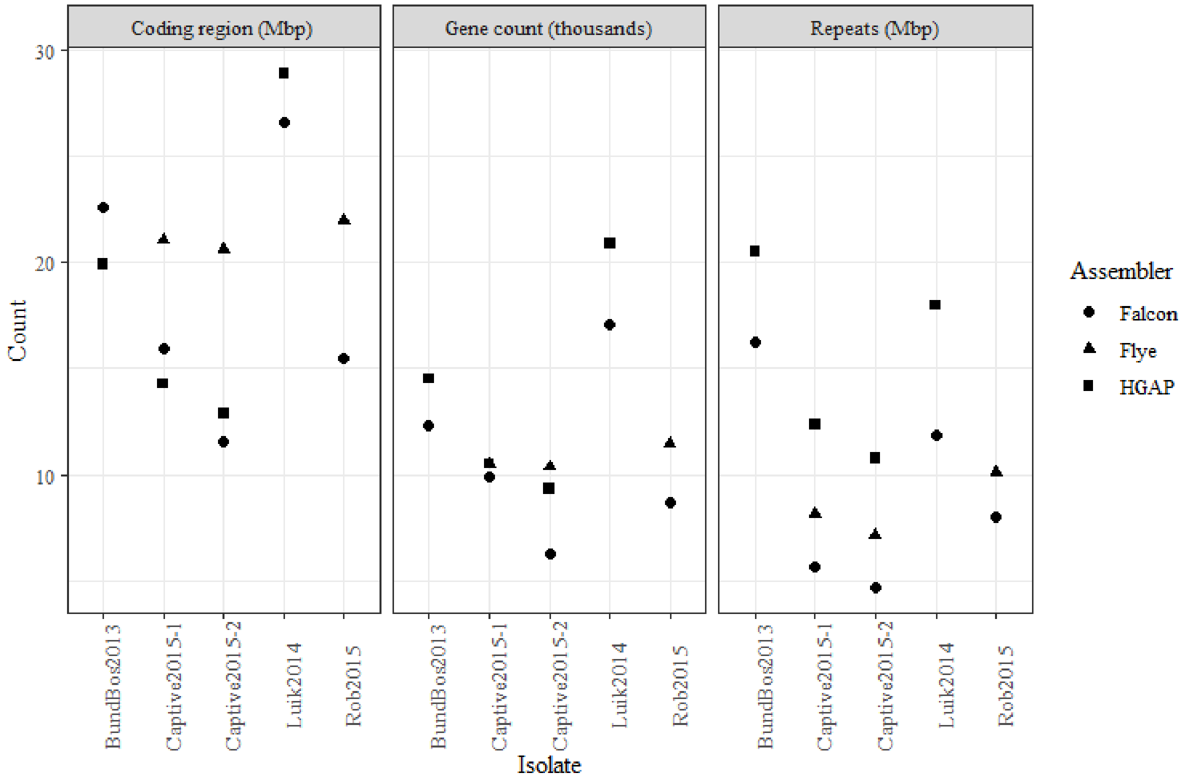

Figure 13. Candidate mitochondrial contigs. Mitochondrial assembly annotated using MITOS[17]

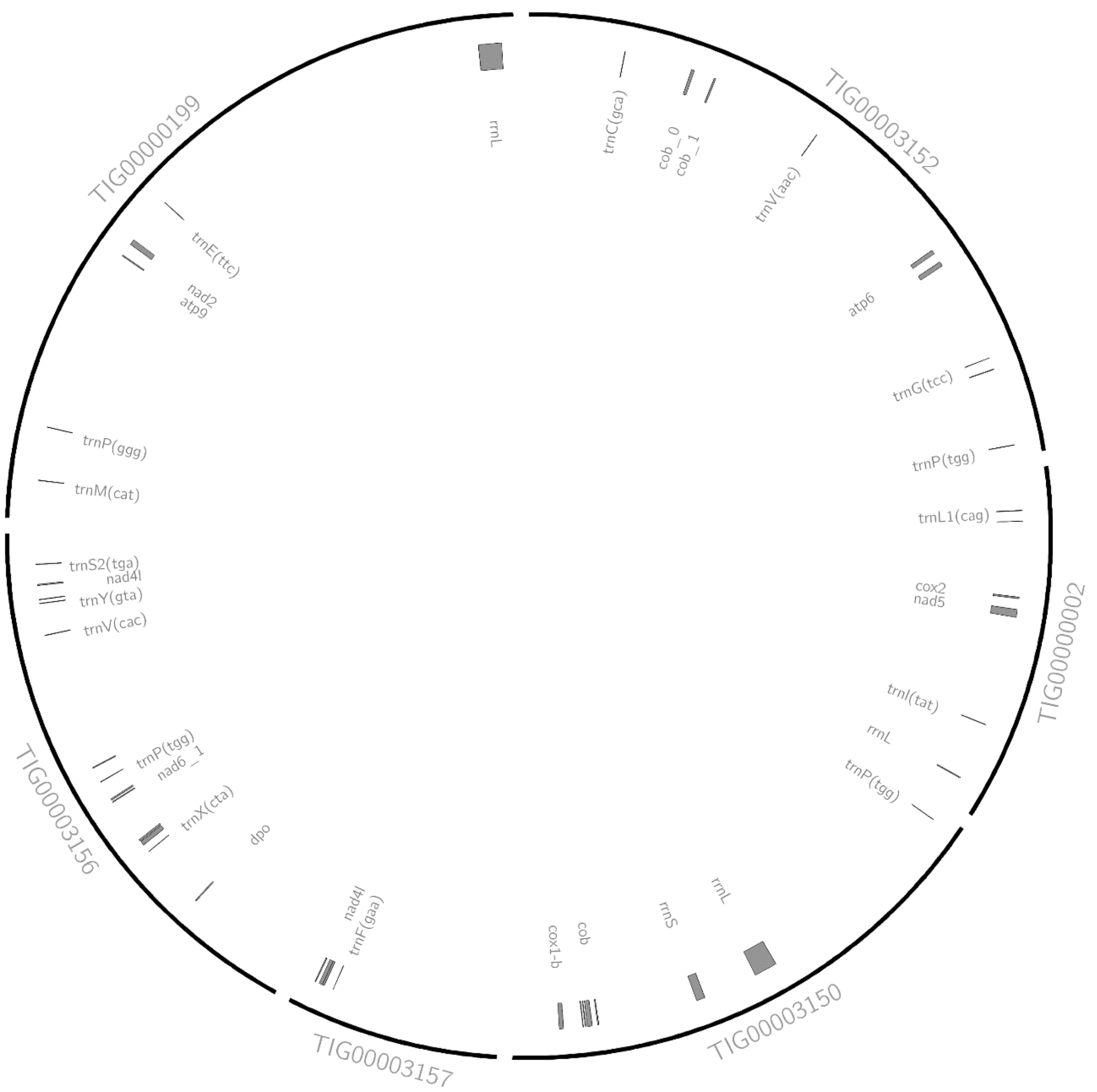

Figure 14. Mitochondrial assembly coverage. Coverage of Rob2015 PacBio reads across candidate mitochondrial contigs. Four significant troughs in coverage represent contig ends.

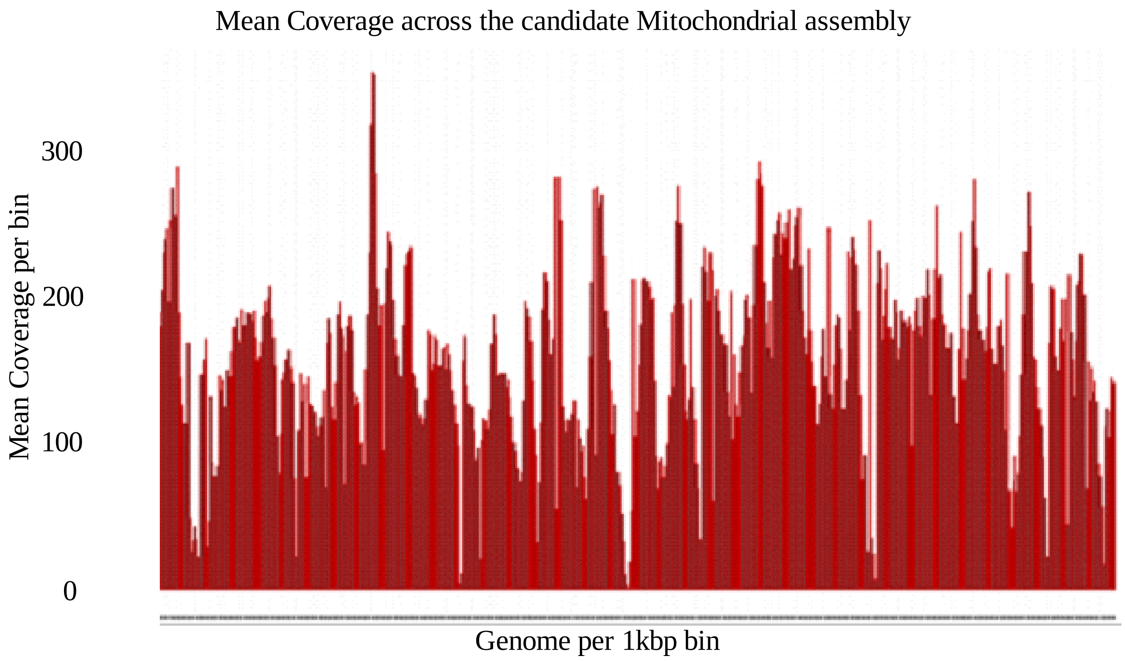

SI Figure 15. Sporangia attachment to various plant material. Boxplots of sporangia attachment at day 4 on a variety of plant material. Boxplots constructed with the centre representing the median, the bounds of the box representing 25th and 75th percentiles, whiskers representing the biggest or smallest value within 1.5 * interquartile range of $25^{th}/75^{th}$ percentiles and data points outside this as outlier points. We see differential attachment to different plant material. Non-autoclaved samples showed considerable contamination at time points greater than 5 days, precluding observation of *Bsal* sporangia attachment. n = 272 (per isolate = 8 wells untreated hay, 8 wells pasteurized leaf litter, 16 wells pasteurized hay and 48 wells autoclaved hay).

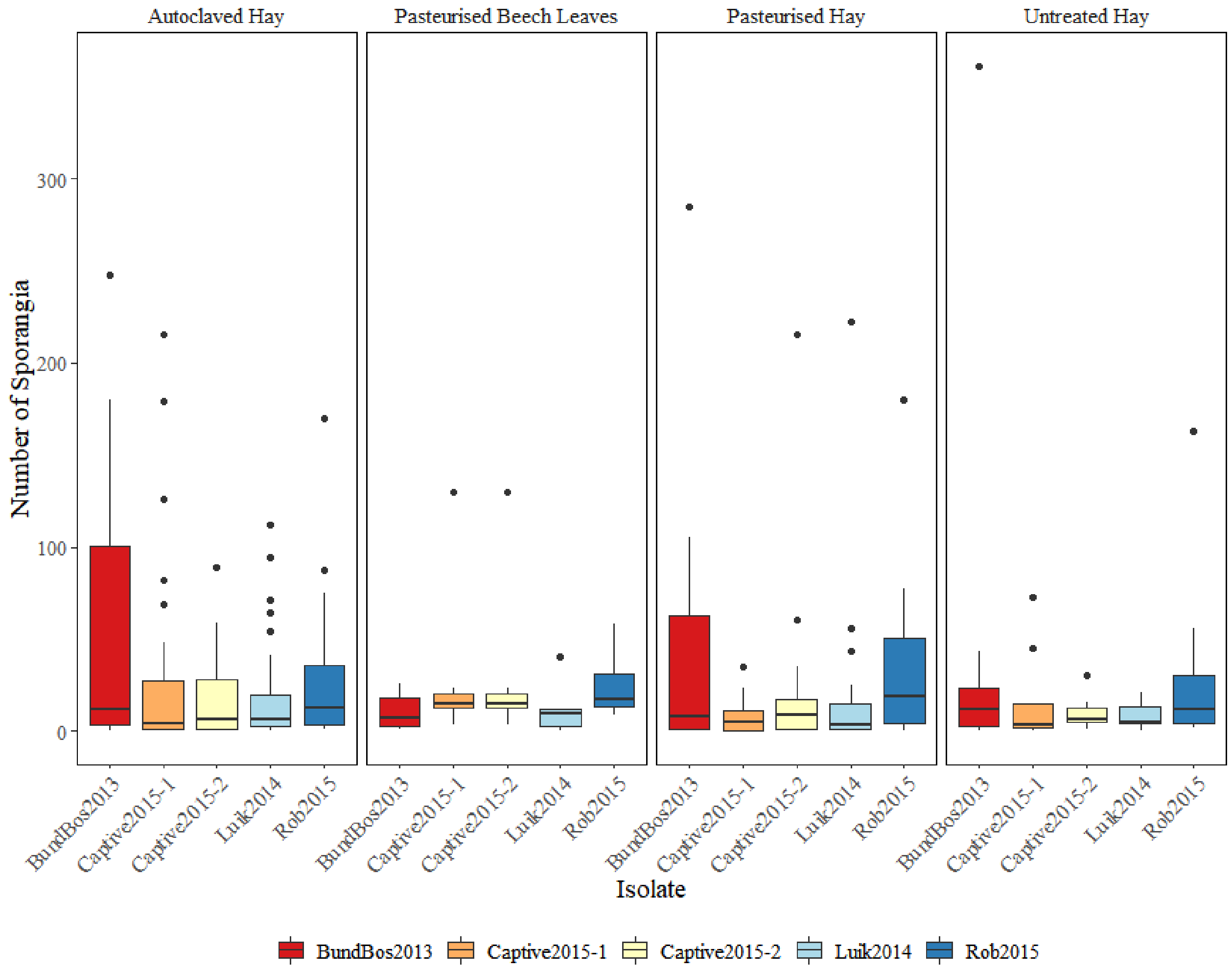

Supplement: Supplementary file 1 — Supplementary Information [file 41467_2021_27005_MOESM1_ESM.pdf]
