# Supplementary material for: Diversity, multifaceted evolution, and facultative saprotrophism in the European Batrachochytrium salamandrivorans epidemic
Source: Nat Commun. 2021 Nov 18;12:6688. doi: 10.1038/s41467-021-27005-0 (PMC8602665; doi:10.1038/s41467-021-27005-0)
Supplement: Supplementary file 3 — Reporting Summary [file 41467_2021_27005_MOESM3_ESM.pdf]

nature research

Corresponding author(s): Moira Kelly, An Martel

Last updated by author(s): Oct 19, 2021

Reporting Summary

Nature Research wishes to improve the reproducibility of the work that we publish. This form provides structure for consistency and transparency in reporting. For further information on Nature Research policies, see our Editorial Policies and the Editorial Policy Checklist.

Statistics

For all statistical analyses, confirm that the following items are present in the figure legend, table legend, main text, or Methods section.

n/a	Confirmed	
☐	☒	The exact sample size (*n*) for each experimental group/condition, given as a discrete number and unit of measurement
☐	☒	A statement on whether measurements were taken from distinct samples or whether the same sample was measured repeatedly
☐	☒	The statistical test(s) used AND whether they are one- or two-sided<br>*Only common tests should be described solely by name; describe more complex techniques in the Methods section.*
☐	☒	A description of all covariates tested
☐	☒	A description of any assumptions or corrections, such as tests of normality and adjustment for multiple comparisons
☐	☒	A full description of the statistical parameters including central tendency (e.g. means) or other basic estimates (e.g. regression coefficient) AND variation (e.g. standard deviation) or associated estimates of uncertainty (e.g. confidence intervals)
☐	☒	For null hypothesis testing, the test statistic (e.g. *F*, *t*, *r*) with confidence intervals, effect sizes, degrees of freedom and *P* value noted<br>*Give P values as exact values whenever suitable.*
☐	☒	For Bayesian analysis, information on the choice of priors and Markov chain Monte Carlo settings
☐	☒	For hierarchical and complex designs, identification of the appropriate level for tests and full reporting of outcomes
☒	☐	Estimates of effect sizes (e.g. Cohen's *d*, Pearson's *r*), indicating how they were calculated

Our web collection on statistics for biologists contains articles on many of the points above.

Software and code

Policy information about availability of computer code

Data collection	no software was used to collect data
Data analysis	SNPrelate version 1.14.0, ggplot2 from tidyr version 0.8.2, glmmADMD version 0.8.3.3, glmmTMD version 0.2.3, betareg version 3.1-3, MASS package, DHARMa package version 0.3.4, extrafont version 0.17, patchwork version 1.1.1, all packages run in R version 3.5<br>RepeatMasker version 4.0.5 with the RepBase version 24.1 fngrep.ref library, BWA version 0.7.12, Picard tools version 1.792, Blast2GO version 2.3.5, HMMER3 with PFAM (release 27) and TIGRFam (release 12) domains, SignalP version 4.0, TMHMM version 2.0, RepeatModeler v1.0.7, HGAP v4 from SMRT Link v5.0 , Falcon Unzip of Falcon 0.5, GAEMR version 1.0.1, Busco v3, Flye 2.4.2 release, MUMmer version 3.23, BLAST v2.2.30, Canu version 1.5, Assemblytics version 1.0, SMRT Tools VariantCaller from SMRT Link v 5.1.0, Samtools v1.9 and Bcftools v1.8, GATK v3.7.93, VCFtools v0.1.10, SNPeff v4.1, Braker, Genemark version 4.31, Augustus version 3.2, RAxML v8.1.15, MUSCLE v3.8.31., SNAPP version 1.42 from BEAST version 2.6.0, OrthoMCL FastTree version 2.1.8, DarkHorse version 2.0, Alienness v.1.0

For manuscripts utilizing custom algorithms or software that are central to the research but not yet described in published literature, software must be made available to editors and reviewers. We strongly encourage code deposition in a community repository (e.g. GitHub). See the Nature Research guidelines for submitting code & software for further information.

Data

Policy information about availability of data

All manuscripts must include a data availability statement. This statement should provide the following information, where applicable:

- Accession codes, unique identifiers, or web links for publicly available datasets
- A list of figures that have associated raw data
- A description of any restrictions on data availability

The raw sequence data have been deposited at GenBank under BioProject PRJNA610831, BioSamples SAMN14316973-SAMN14316981 and SAMN17104240-

SAMN17104244, and SRA objects SRR11252126-SRR11252134. Other data referenced in this paper are outlined in the supplementary information.
Other publicly available data used in this manuscript:
MEROPS “pepunit.lib” database containing 1,103,662 proteases (downloaded from http://merops.sanger.ac.uk/ 22 April 2018),
Carbohydrate-Active enZYmes Database CAZyDB.07202017 file (http://www.cazy.org/, downloaded 7th June 2018 containing 921,174 protein sequences), t
he BBatrachochytrium dendrobatidis Jel423 assembly (Bioproject PRJNA13653, Accession GCA_000149865.1, https://www.ncbi.nlm.nih.gov/assembly/GCA_000149865.1/),
the Batrachochytrium salamandrivorans BundBos2013 assembly (Bioproject PRJNA311566, Accession GCA_002006685.1, https://www.ncbi.nlm.nih.gov/assembly/GCA_002006685.1/)
NCBI blast nr database, downloaded 16th August 2018 (https://ftp.ncbi.nlm.nih.gov/blast/db/nr*)

Field-specific reporting

Please select the one below that is the best fit for your research. If you are not sure, read the appropriate sections before making your selection.

☐ Life sciences ☐ Behavioural & social sciences ☒ Ecological, evolutionary & environmental sciences

For a reference copy of the document with all sections, see nature.com/documents/nr-reporting-summary-flat.pdf

Ecological, evolutionary & environmental sciences study design

All studies must disclose on these points even when the disclosure is negative.

Study description	The study involves the genomic and phenotypic analysis of nine isolates of Batrachochytrium salamandrivorans fungi. Phenotypic tests involved treatments of different nutritional sources- complex plant material of autoclaved or pasteurised hay, straw, beech leaf (Fagus sylvatica), lima bean (Phaseolus lunatus) medium with a TGhL (tryptone, gelatin hydrolysate and lactose) medium positive control and a heat-killed or sham-treated (sterile distilled water) negative control. Treatments of complex vegetative material was nested per plant sample per experiment.
Research sample	Nine isolates of Batrachochytrium salamandrivorans , isolated from four wild outbreak sites (two isolates were serially collected from three outbreak sites 1-5.5 years apart) and two outbreaks in captive amphibian collections. We aimed to get a sample representative of the European Batrachochytrium salamandrivorans outbreak. Our sample size was limited by the number of strains that had been successfully isolated at the time of the study - the difficulties involved in successfully isolating Bsal, meant that only 11 isolates had been successfully isolated for this species (known by the authors) at the onset for this study. Sufficient genomic DNA was isolated from nine of these, which covered the geographic and temporal range of the known European Bsal epidemic, and so these were sequenced.
Sampling strategy	Batrachochytrium salamandrivorans strains were isolated from infected fire salamanders (Salamandra salamandra), or a marbled newt (Triturus marmoratus) for the Catalan2018 isolate, naturally infected during their respective outbreak. Isolates were collected using protocols as described in Martel et al. (2013); 1-2mm skin sections are dragged through agar medium to remove surface contamination, before being placed in TGhL (tryptone, gelatin hydrolysate and lactose) medium with 200 mg/L penicillin-G and 400 mg/L streptomycin sulphate antibiotics and incubated at 15 °C. At the onset of the study, a total of 11 strains had been isolated from this species (known the by the authors). These were the nine strains for which sufficient genomic material could be successfully gathered using the yeast protocol for Qiagen 100G genomic tips. For phenotypic experiments, sample sizes were selected based on power analyses from preliminary experiments, and temporal estimates from these preliminary experiments- such that all observations per time point could be completed within 6 hours so that observations remained comparable as having been performed at the same time point.
Data collection	Data collection was completed by the first author, observational data was initially noted manually with a pen and paper and then entered into spreadsheets. Samples (cultures in flasks or wells) were visualised with an inverted microscope (Nikon Eclipse TS100, Nikon Instruments). Data collection for phenotypic tests involved visual observations- these were performed blinded. Samples were only unblinded after experiments were completed and raw data saved in excel sheets.
Timing and spatial scale	Batrachochytrium salamandrivorans isolate collection is ongoing- since it's discovery in 2013 the authors of this paper (AM and FP) have attempted to isolate strains of B. salamandrivorans from all outbreaks known to them. Isolates collected before January 2019, for which adequate genomic material could be gathered were included in this manuscript. The rational for performing this analysis (including isolates up until 2019), was that as only 1 or 2 strains are successfully isolated per year, waiting for a meaningfully larger sample size of isolates would take many years, delaying the release of this information. While our sample size is small, it does cover the known geographical range of the European B. salamandrivorans outbreaks and is a substantial improvement upon the single sample for which genomic and phenotypic data is currently published.<br><br>For mixed vegetative phenotypic experiments, data was collected every five days for 25 days, as five days represents the generation time for Batrachochytrium salamandrivorans. For the beech leaf material growth comparison of AMFP14/2 and AMFP18/1 data was collected every 10 days for 30 days, given the normal 5-day replication cycle of Batrachochytrium salamandrivorans, we estimated that this time frame represented two generations between each data collection point. All laboratory experiments were performed between August 2018 and September 2019.
Data exclusions	No experimental data was excluded from analysis, but for preliminary experiments involving non-autoclaved plant material, contamination of samples and bacterial overgrowth sometimes precluded data collection- such samples were removed from the experiment and this limited the analysis of these data and led to an adaptation of the protocol to only include autoclaved plant material for longer growth-monitoring experiments- as detailed in the methods section.

Reproducibility	All experiments were repeated independently at least twice, all repetitions were successful and were included in this manuscript. The finding of a vegetative metabolic capacity is an infrequent event and so there is variability between experiment results however all experiments indicated some capacity in isolates AMFP15/3, AMFP14/2 and AMFP13/1, and in no experiments was vegetative growth observed in isolates AMFP15/1 or AMFP15/2- this is discussed in the manuscript.
Randomization	Studies were blinded, but at no point were isolates grouped for analysis, all isolates were assessed for growth on a section from the same plant samples- with results nested per plant sample, and so there was no need for randomisation.
Blinding	All experiments were blinded- the data collector was unaware of which samples represented each isolate throughout the experiments and data logging, although the positive control was visibly distinguishable due to the nature of the control. For each experiment, the researcher was blinded at the point of analysis (visualisation and model fitting) of phenotypic data, data was unblinded when combining experiments and figure generation.

Did the study involve field work? ☐ Yes ☒ No

Reporting for specific materials, systems and methods

We require information from authors about some types of materials, experimental systems and methods used in many studies. Here, indicate whether each material, system or method listed is relevant to your study. If you are not sure if a list item applies to your research, read the appropriate section before selecting a response.

Materials & experimental systems

n/a	Involved in the study
☒	☐ Antibodies
☒	☐ Eukaryotic cell lines
☒	☐ Palaeontology and archaeology
☒	☐ Animals and other organisms
☒	☐ Human research participants
☒	☐ Clinical data
☒	☐ Dual use research of concern

Methods

n/a	Involved in the study
☒	☐ ChIP-seq
☒	☐ Flow cytometry
☒	☐ MRI-based neuroimaging

Animals and other organisms

Policy information about studies involving animals; ARRIVE guidelines recommended for reporting animal research

Laboratory animals	The study did not involve laboratory animals.
Wild animals	The study did not involve any wild animals.
Field-collected samples	The study did not involve field-collected samples.
Ethics oversight	No ethical approval was required as all data and experiments only involved plants and single cell fungal organisms.

Note that full information on the approval of the study protocol must also be provided in the manuscript.
