# Supplementary material for: Diversity, multifaceted evolution, and facultative saprotrophism in the European Batrachochytrium salamandrivorans epidemic
Source: Nat Commun. 2021 Nov 18;12:6688. doi: 10.1038/s41467-021-27005-0 (PMC8602665; doi:10.1038/s41467-021-27005-0)
Supplement: Supplementary file 4 — Description of Additional Supplementary Files [file 41467_2021_27005_MOESM4_ESM.pdf]

Description of Additional Supplementary Files

Supplementary Data 1:
MEROPs protease and CAZyme candidate counts per isolate, listing enzyme family and type enzyme.

Supplementary Data 2:
BundBos isolate copy number variation (CNV): Genes identified as having varied copy number and Pfams indicated as enriched in Copy Number Variation (CNV) enrichment tests, comparing BundBos2013 and BundBos2018. Contains genes, Pfam annotations, copy number ratio and adjusted p values

Supplementary Data 3:
Horizontal Gene Transfer (HGT) candidates per isolate, noting Pfam annotation, Alienness AI score, Darkhorse LPI score and best hit identified by Darkhorse 2
